# Detecting infrared UAVs on edge devices through lightweight instance segmentation

**DOI:** 10.1371/journal.pone.0330074

**Published:** 2025-08-18

**Authors:** YuZhi Chen, HaoYue Sun, Liang Tian, Ye Yang, ShenYang Wang, TianYou Wang

**Affiliations:** 1 Hebei University of Architecture, Zhangjiakou, China; 2 Hebei Digital Education Collaborative Innovation, Shijiazhuang, China; 3 College of Computer and Cyber Security, Hebei Normal University, Shijiazhuang, China; Kafkas University: Kafkas Universitesi, TÜRKIYE

## Abstract

**Motivation:**

Infrared unmanned aerial vehicle (UAV) detection for surveillance applications faces three conflicting requirements: accurate detection of pixel-level thermal signatures, real-time processing capabilities, and deployment feasibility on resource-constrained edge devices. Current deep learning approaches typically optimize for one or two of these objectives while compromising the third.

**Method:**

This paper presents YOLO11-AU-IR, a lightweight instance segmentation framework that addresses these challenges through three architectural innovations. First, Efficient Adaptive Downsampling (EADown) employs dual-branch processing with grouped convolutions to preserve small-target spatial features during multi-scale fusion. Second, HeteroScale Attention Network (HSAN) implements grouped multi-scale convolutions with joint channel-spatial attention mechanisms for enhanced cross-scale feature representation. These architectural optimizations collectively reduce computational requirements while maintaining detection accuracy. Third, Adaptive Threshold Focal Loss (ATFL) introduces epoch-adaptive parameter tuning to address the extreme foreground-background imbalance inherent in infrared UAV imagery.

**Results:**

YOLO11-AU-IR is evaluated on the AUVD-Seg300 dataset, achieving 97.7% mAP@0.50 and 75.2% mAP@0.50:0.95, surpassing the YOLO11n-seg baseline by 1.7% and 4.4%, respectively. The model reduces parameters by 24.5% and GFLOPs by 11.8% compared to YOLO11n-seg, while maintaining real-time inference at 59.8 FPS on an NVIDIA RTX 3090 with low variance. On the NVIDIA Jetson TX2, under INT8 CPU-only deployment, YOLO11-AU-IR retains 95% mAP@0.50 with minimal memory footprint and stable performance, demonstrating its practical edge compatibility. Ablation studies further confirm the complementary contributions of EADown, HSAN, and ATFL in enhancing accuracy, robustness, and efficiency. Code and dataset are publicly available at https://github.com/chen-yuzhi/YOLO11-AU-IR.

## 1. Introduction

Unmanned aerial vehicles (UAVs) have rapidly advanced in various applications such as environmental monitoring, infrastructure inspection, and logistics due to their flexibility and cost-effectiveness. However, the rapid proliferation of UAV technology has raised concerns about its misuse in unauthorized surveillance, environmental monitoring, and other public safety challenges. This highlights the critical need for robust and efficient UAV detection technologies, particularly in real-time and resource-constrained environments, such as public safety and environmental monitoring [1]. Among the various detection methods, visual-based detection has garnered significant research interest due to its precision, ease of data acquisition, and intuitive visual outputs [2]. Infrared sensors, in particular, offer advantages over visible-light sensors, especially in complex environments such as low-light or adverse weather conditions [3].

**Fig 1:**
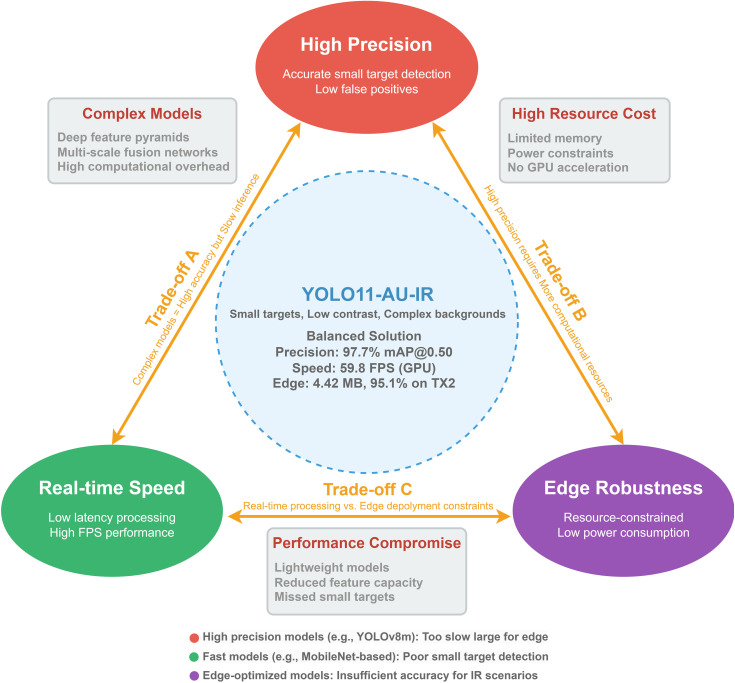
IR UAV Detection Trilemma.

IR-based UAV detection faces three interconnected challenges that we term the “IR UAV Detection Trilemma” (Fig 1). In practice, detection systems struggle to simultaneously achieve: (1) accurate detection of small targets, (2) real-time processing speed, and (3) deployment on resource-limited devices. Current approaches typically excel at one or two aspects while compromising the third.

Specifically, these challenges manifest as:

1. High Precision: Accurately detecting small, low-contrast UAVs in noisy IR imagery while minimizing false alarms. The inherent low thermal contrast between UAVs and their backgrounds, combined with sensor noise, creates reduced feature dimensionality and blurred target boundaries. This makes detecting small or distant UAVs particularly challenging, as they may appear as mere pixels indistinguishable from noise [4].
2. Real-time Speed: Processing video streams fast enough for immediate threat response and tracking. Current deep learning models struggle to balance computational demands with speed requirements. While complex architectures with multi-scale feature pyramids achieve high accuracy, they cannot meet the stringent latency requirements for real-time UAV tracking. Conversely, streamlined models that achieve acceptable framerates often sacrifice the sophisticated feature extraction necessary for reliable small-target detection [5].
3. Edge Robustness: Operating reliably on resource-constrained devices across varying environmental conditions. Practical deployment scenarios require detection systems to run on edge devices with limited computational power and strict energy budgets. This creates a fundamental tension: models must be lightweight enough for embedded systems while maintaining sufficient representational capacity to handle diverse IR scenarios, from thermal inversions to cluttered backgrounds [6].

Current approaches typically excel in one or two aspects while compromising the third. High-precision models are too computationally intensive for edge deployment, fast models miss small targets due to oversimplified architectures, and robust models require resources beyond what edge devices can provide. The primary contributions of this work are summarized as follows:

- **Construction of AUVD-Seg300.** A specialized IR UAV dataset annotated with pixel-level segmentation masks. This dataset addresses IR imaging challenges by capturing precise geometric boundary information, thereby mitigating the feature degradation common in IR imagery.
- **Efficient Adaptive Downsampling (EADown).** A novel downsampling module that preserves key fine-grained features of low-signal targets while reducing resolution, making it particularly suitable for small-scale datasets.
- **HeteroScale Attention Network (HSAN).** A multi-scale attention mechanism that enhances feature representation by enabling cross-scale interaction and dynamic feature adaptation, improving detection across varying UAV sizes. The HSAN enhances feature representation by dynamically adapting to different scales of UAVs, improving detection accuracy for small targets in complex IR environments.
- **Adaptive Threshold Focal Loss (ATFL).** A dynamic loss function that balances the model's attention between difficult and easy-to-classify samples, mitigating the problem of class imbalance and enhancing the model's robustness. The ATFL addresses class imbalance by dynamically focusing on difficult samples, improving the model's robustness without sacrificing real-time performance.
- **Comprehensive Experimental Validation.** Experiments on AUVD-Seg300 indicate that YOLO11-AU-IR achieves 97.7% mAP@0.50 and 75.2% mAP@0.50:0.95, using only 4.42 MB of model storage at 59.8 FPS on a high-end GPU. Furthermore, deployment on an NVIDIA Jetson TX2—under CPU-only, INT8 quantization—demonstrates the model's feasibility in low-power scenarios, retaining a 95.1% mAP@0.50.

By demonstrating high-precision small-target detection at real-time speeds in IR imagery, YOLO11-AU-IR addresses the tri-lemma of precision, speed, and robustness. The architecture's modular design also allows straightforward adaptation to other scenarios involving small, faint objects in thermally variable environments, such as public safety and environmental monitoring. In the following section, we review relevant literature, focusing on YOLO‐based UAV detection and IR‐specific challenges. In the subsequent section, we introduce our AUVD‑Seg300 dataset with pixel‐level IR UAV annotations. Furthermore, we detail our model design, including dataset construction and the EADown, HSAN, and ATFL modules. We then report extensive experimental comparisons and ablation results before discussing edge‐deployment performance. Finally, we summarize our contributions and outline future research directions, including multi‐sensor data fusion and more extensive field tests.

## 2. Related work

Infrared UAV detection faces three fundamental challenges: extremely small target representation with UAVs often appearing as fewer than 100 pixels, low thermal contrast between UAVs and backgrounds particularly during thermal crossover periods, and high noise levels inherent to infrared sensors that further degrade already-weak signals. These core challenges have driven the development of various detection methodologies, each offering distinct advantages and limitations.

UAV detection technologies predominantly comprise radar-based, acoustic, radio frequency (RF), and vision-based approaches, each with distinct operational contexts, advantages, and limitations [7]. Radar-based detection exhibits reduced sensitivity to adverse weather conditions and illumination variations; however, it is constrained by the minimal radar cross-section of micro-UAVs and frequently demonstrates inadequate precision in complex environmental contexts. Acoustic detection presents cost-effective implementation and can partially differentiate between UAVs and avian species, yet remains susceptible to ambient noise interference and offers limited detection range. RF detection, while facilitating straightforward deployment and enabling remote UAV control and localization, exhibits elevated failure risk in environments characterized by unknown frequency bands or radio silence, and demonstrates vulnerability to meteorological conditions and signal interference.

Vision-based UAV detection has emerged as the most mature approach, offering intuitive visual feedback and straightforward data acquisition for practical applications [8]. While conventional visible-spectrum systems struggle in low-light conditions, infrared imaging technology penetrates atmospheric obscurants and maintains effectiveness in darkness [9]. However, infrared imagery typically exhibits lower spatial resolution than visible-spectrum imagery, resulting in reduced target detail. Combined with increased susceptibility to noise interference, these factors significantly increase detection complexity [10].

### 2.1. Datasets

The development of effective UAV detection models depends heavily on high-quality, diverse training datasets. Table 1 summarizes representative publicly available datasets, each with specific strengths and limitations.

**Table 1:** Related datasets.

Datasets	Description	Limitations
**GA-Fly [12]**	Specializes in small-target UAV detection, comprising 10,800 high-definition images encompassing multiple angular perspectives and illumination conditions.	Limited to a single UAV model, in-sufficient environmental diversity, absence of infrared data.
**VisioDECT Dataset [13]**	Encompasses 20,924 samples of six UAV variants across diverse scenarios, providing multiple annotation formats.	Predominantly focused on visible-spectrum imagery, insufficient infrared data support.
**Anti-UAV [14]**	High-resolution video sequences of multi-scale UAVs, incorporating both RGB and IR modalities, applicable to dynamic scenarios.	Substantial data volume necessitating significant computational resources, lacking specialized optimization for small-target segmentation.
**Drone-detection-dataset [15]**	Multi-modal database integrating visible-spectrum, infrared, and audio data.	Insufficient coverage of nocturnal scenarios, limited infrared data volume.
**Drone-dataset [16]**	Approximately 1,400 UAV images extracted from web-based sources.	Heterogeneous quality, absence of infrared or dynamic scenario data.
**DUT Anti-UAV [17]**	Extensive resolution range, detection subset comprising 10,000 images.	Relatively constrained target quantity, significant image resolution disparity, inconsistent annotation precision.
**ARD-MAV [18]**	Incorporates 60 video sequences encompassing 106,665 frames, complex and heterogeneous scenarios, minimal average target area proportion.	Limited to a single UAV model, in-sufficient dynamic characteristics, reduced adaptability to high-velocity targets.

Current datasets exhibit several critical issues. First, most remain predominantly visible-spectrum focused, with infrared imagery comprising only a small fraction, limiting model adaptation to nocturnal scenarios. Second, insufficient representation of small targets constrains model performance when UAVs occupy minimal pixel areas. Third, limited diversity in UAV types and thermal signatures restricts generalization capabilities. Finally, most datasets employ bounding box annotations, while cost-effective, provide insufficient precision for infrared small-target detection. Research indicates that segmentation-based annotations offer enhanced spatial precision, contributing to improved boundary delineation and detection accuracy [11].

We construct the AUVD-Seg300 dataset which addresses these limitations through pixel-level segmentation masks for infrared imagery, enabling fine-grained boundary extraction even for targets reduced to pixel-level spots [19]. This approach captures morphological details critical for distinguishing UAVs from background noise in low-contrast thermal environments.

### 2.2. YOLO-based vision approaches for UAV detection

The YOLO architecture has become the dominant framework for real-time UAV detection, with numerous adaptations addressing infrared-specific challenges. Table 2 chronicles the evolution of YOLO-based methods from 2020 to 2024, revealing three main areas of innovation: backbone modifications, neck architecture improvements, and head network optimizations.

**Table 2:** YOLO-Based Visual UAV Detection Methods.

Ref.	Year	Base Model
[20]	2020	YOLOv4
[21]	2021	YOLOv3
[22]	2021	YOLOv3
[23]	2021	YOLOv4
[24]	2021	YOLOv4
[17]	2022	YOLOX
[25]	2022	YOLOv5s
[26]	2022	YOLOv3
[27]	2022	YOLOv5
[28]	2023	YOLOv4
[29]	2023	YOLOv4
[30]	2023	YOLOvX-nano
[31]	2024	YOLOv8
[32]	2024	YOLOv7-tiny

Backbone modifications have prioritized lightweight architectures suitable for edge deployment. Cheng et al. [29] integrated MobileViT into YOLOv4, achieving 92.81% mAP with only 13.47M parameters through efficient local-global feature representation. Wang et al. [30] pushed efficiency further using depth-separable convolutions in YOLOX, compressing the model to 3.85MB while maintaining 82.32% mAP. For scenarios requiring higher accuracy, Bo et al. [32] introduced InceptionNeXt modules in YOLOv7-GS, specifically targeting low-altitude UAVs in cluttered backgrounds.

Neck architectures have evolved to better handle multi-scale targets. Early work by Singha and Aydin [23] utilized standard PANet, while subsequent research explored more sophisticated fusion strategies. Lv et al. [25] balanced speed and accuracy through Ghost modules and SimAM attention in SAG-YOLOv5s. Cheng et al. [29] enhanced positional awareness via Coordinate Attention in their CA-PANet module. Most recently, Bo et al. [32] introduced SPPFCSPC-SR modules to preserve small-target features during multi-scale fusion.

Head network optimizations focus on precise localization in challenging infrared conditions. Zhao et al. [17] established the importance of optimized decoding layers on the DUT Anti-UAV dataset. Building on this, Lv et al. [25] implemented α-DIoU loss functions for improved bounding box regression, achieving 97.6% precision. Huang et al. [30] integrated DCNv2 for a 3.1% mAP improvement in complex backgrounds, while Bo et al. [32] developed Get-and-Send modules specifically for small-target attention.

Recent lightweight YOLO variants employ various attention mechanisms: YOLOv7-tiny uses polarization self-attention, while MobileViT-YOLO leverages transformer-based attention. However, these mechanisms process features at uniform scales, unsuitable for infrared UAVs that appear as pixel-sized heat signatures at varying distances. Similarly, existing loss functions like VFL and DFL in YOLOv8 use static parameters throughout training, failing to adapt to the evolving difficulty of infrared small-target detection.

Performance evaluations across these methods demonstrate consistent progress toward balancing accuracy and efficiency. Through targeted architectural modifications and novel feature fusion strategies, YOLO-based methods have achieved satisfactory detection performance despite the constraints of infrared imaging.

### 2.3. YOLO-based detection methods for edge devices

Edge deployment presents unique challenges for UAV detection systems, requiring optimization across computational efficiency, power consumption, and real-time performance. Edge devices—including embedded systems like Raspberry Pi and NVIDIA Jetson platforms—offer mobility and low power consumption but impose strict constraints on model complexity [33–35].

Edge computing relocates data processing closer to sources, minimizing network latency while enhancing privacy protection [36]. For UAV detection, this enables real-time inference without cloud dependency, critical for responsive threat detection. The continuous evolution of YOLO architectures has produced lightweight variants specifically targeting these constraints [37].

Algorithm optimization strategies include channel pruning based on importance metrics, removing filters with minimal feature contribution [38]. Quantization from FP32 to FP16 or INT8 precision reduces memory usage and accelerates inference, particularly when combined with hardware acceleration libraries like TensorRT or OpenVINO [39]. Replacing standard backbones with lightweight alternatives such as MobileNet or ShuffleNet further reduces computational requirements [30,40].

Hardware adaptation requires coordinated utilization of heterogeneous computing resources. On platforms like NVIDIA Jetson, CPUs handle preprocessing and control logic while GPUs focus on convolution operations. Pipeline design and asynchronous queues maximize resource utilization by enabling parallel processing [41]. Intel platforms offer VPU or FPGA acceleration through OpenVINO, providing flexible inference capabilities [42].

### 2.4. Summary

Current vision-based UAV detection research has made substantial progress in architectural design, yet infrared-specific challenges remain largely unresolved. While innovations in lightweight backbones, multi-scale fusion, and attention mechanisms have improved general detection performance, these advances often fail to address the unique characteristics of infrared imaging: severely limited pixel representation of distant UAVs, dynamic thermal contrast variations, and the absence of color or texture cues that visible-spectrum methods rely upon.

The scarcity of high-quality infrared datasets with pixel-level annotations represents the most critical bottleneck. Without sufficient training data that captures the full spectrum of thermal signatures, atmospheric conditions, and UAV types, even sophisticated architectures struggle to generalize effectively. This limitation is particularly acute for small UAVs that may occupy fewer than 100 pixels in typical surveillance scenarios. Consequently, developing specialized modules for small-target feature preservation, adaptive loss functions for extreme class imbalance, and efficient architectures for edge deployment becomes essential.

## 3. Dataset construction

The development of effective infrared UAV detection models requires high-quality datasets with precise annotations. Existing datasets predominantly focus on visible-spectrum imagery or provide only bounding box annotations, which are insufficient for pixel-level segmentation of small thermal targets. To address this gap, we constructed the AUVD-Seg300 dataset, providing the first publicly available infrared UAV dataset with pixel-level segmentation masks.

Our dataset is derived from the Anti-UAV benchmark [14] infrared video sequences, proposed by the Beijing Institute of North Electronic Equipment. The Anti-UAV dataset includes high-definition video sequences (both RGB and IR) capturing various UAV types across diverse environments including daylight, nighttime, clouds, buildings, forests, and potential interference sources such as birds and airborne debris.

For our study, we extracted IR video clips (each 50 seconds in duration at 30 FPS) and implemented a systematic sampling strategy (one frame extracted every 16 seconds, approximately 3 frames per video). This process yielded 533 usable infrared images. Following careful examination, we selected and annotated 300 images with pixel-level segmentation masks using the Labelme [43] tool, following established practices for image annotation in computer vision tasks [44]. The dataset was carefully curated to include equal representation of three UAV platform categories (100 images each):

- Large UAVs: Industrial multi-rotor platforms with 6 or more rotors and diagonal wheelbase exceeding 600mm
- Normal UAVs: Standard consumer quad-copters with diagonal wheelbase between 250-600mm
- Tiny UAVs: Micro/mini drones with diagonal wheelbase under 250mm

This categorization reflects real-world security scenarios where different UAV types pose varying levels of threat and detection difficulty. Large UAVs, while having stronger thermal signatures, often operate at greater distances; Normal UAVs balance detect-ability with operational flexibility; and Tiny UAVs present the greatest challenge due to minimal heat emission and small physical profiles.

**Fig 2:**
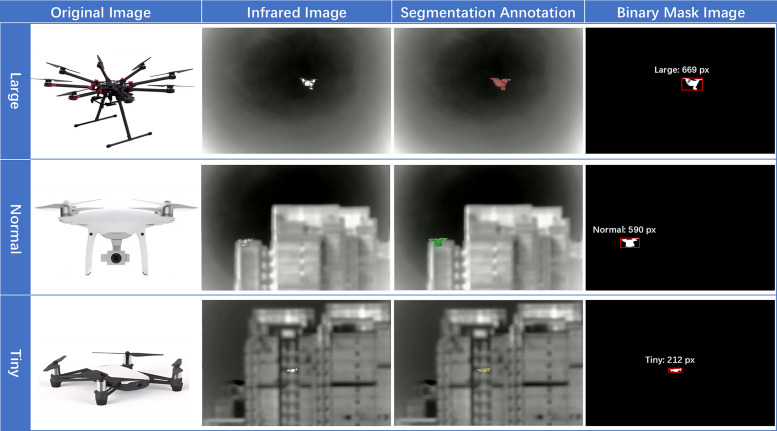
Infrared UAV Dataset (AUVD-Seg300) and Segmentation Annotations.

Representative examples from each UAV category are illustrated in (Fig 2), which contrasts the original visible-light references with their infrared versions, all annotated at the pixel level. The resulting AUVD-Seg300 dataset was randomly partitioned into training, validation, and testing sets using a 6:2:2 ratio, ensuring balanced representation of UAV types across all splits. This dataset addresses the critical need for pixel-level annotations in infrared small-target detection, enabling precise boundary extraction even for targets reduced to pixel-level thermal spots.

## 4. Methodology

**Fig 3:**
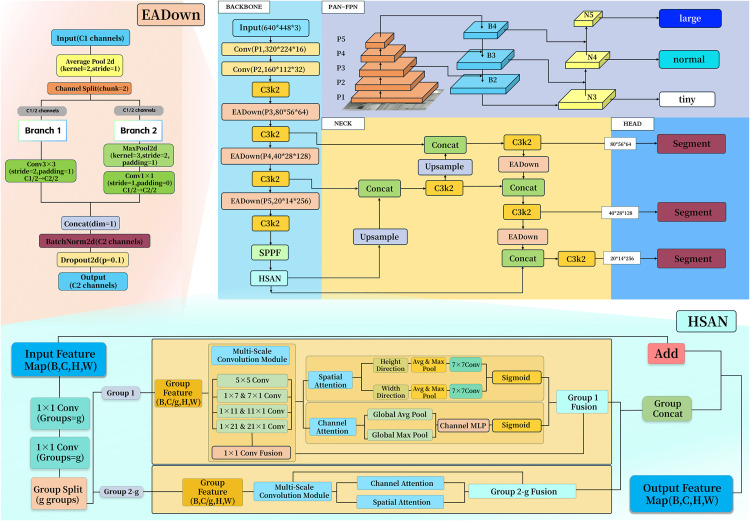
Detailed technical architecture of YOLO11-AU-IR.

This section details the proposed YOLO11-AU-IR framework, highlighting its key architectural designs for infrared small-target UAV detection and segmentation. The overall structure and information flow of the model are illustrated in (Fig 3).

### 4.1. Base model selection

We selected YOLO11n [45] as our base architecture based on its recent release and architectural advantages for lightweight instance segmentation. This choice was driven by three key factors:

1. Balanced Architecture for Instance Segmentation: YOLO11n represents the latest evolution in the YOLO series, incorporating architectural improvements that balance model size with segmentation performance. With 2.84M parameters (as shown in Table 5), it provides an optimal foundation for further optimization while maintaining sufficient capacity for infrared UAV detection.
2. Lightweight Design: Among the segmentation models evaluated in our experiments, YOLO11n-seg demonstrates competitive baseline performance (96.0% mAP@0.50, 70.8% mAP@0.50:0.95) while maintaining a compact size of 5.7MB. This provides adequate headroom for our architectural enhancements without exceeding edge deployment constraints.
3. Modular Architecture: YOLO11n's structure facilitated the integration of our proposed modules (EADown, HSAN, ATFL). The clear separation between backbone, neck, and head components allowed targeted modifications at specific network stages, enabling us to replace standard modules with our optimized alternatives while preserving the overall architectural integrity.

**Table 3:** Module replacement computational cost analysis.

Original Module	GFLOPs	New Module	GFLOPs	Reduction
**Conv**	34.49	EADown	21.54	37.5%
**C2PSA**	129.7	HSAN	29.29	77.4%

**Table 4:** Hardware and software platform configurations.

Configuration	Version Parameter
**GPU**	RTX 3090(24GB)
**CPU**	Intel(R) Xeon(R) Platinum 8362 CPU @ 2.80GHz
**CUDA**	11.8
**Python**	3.8.10
**Deep learning framework**	PyTorch 2.0.0

**Table 5:** a. Comparative results of lightweight models (≤15MB) on AUVD-Seg300 dataset. b. Comparative results of medium and heavyweight models (>15MB) on AUVD-Seg300 dataset.

Model	Parameters	GFLOPs	FPS	P	R	mAP@0.50(%)	mAP@0.50:0.95(%)	Size(MB)
**yolov5n-seg**	1882456	6.7	117.78 ± 7.63	62.6	75.4	69.9	43.4	3.89
**yolov5s-seg**	7403816	25.7	131.15 ± 14.16	94.4	82.1	95.7	64.1	14.4
**yolov5m-seg**	21660440	69.9	105.01 ± 7.4	90.4	88.5	94.9	67.3	41.7
**yolov7-seg**	37853264	141.9	73.06 ± 4.94	93.8	97.7	97.6	71.1	76.2
**yolov8n-seg**	3258649	12	69.65 ± 2.48	95.2	92.1	96.2	72.1	6.45
**yolov8s-seg**	11780761	42.4	61.99 ± 1.55	95.3	96.6	97.4	73.6	22.7
**yolov8m-seg**	27224121	110	56.66 ± 4.12	97.2	96.2	97.9	74.9	52.2
**YOLO-mamba-seg**	6237585	17.5	53.05 ± 1.37	95.0	89.7	94.5	70.4	12.8
**yolo11n-seg**	2835153	10.2	63.91 ± 5.75	95.6	88.8	96.0	70.8	5.7
**Ours**	**2139591**	**9**	**59.8 ± 0.74**	**96.7**	**97.0**	**97.7**	**75.2**	**4.42**

* Results represent mean ± standard deviation over 5 independent training runs. FPS results represent mean ± standard deviation from multiple inference runs on RTX 3090 (batch size 16).

While other lightweight models like YOLOv5n-seg (3.89MB) offer smaller sizes, they show significantly lower baseline performance (69.9% mAP@0.50). Conversely, larger models like YOLOv8n-seg achieve slightly better baseline results (96.2% mAP@0.50) but require 6.45MB storage—limiting the optimization space for edge deployment. YOLO11n thus provides the optimal starting point for developing a lightweight, edge-deployable infrared UAV detection system.

### 4.2. Framework overview

**Fig 4:**
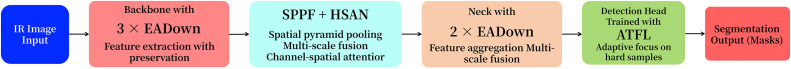
Simplified flow diagram highlighting the integration of our key contributions (EADown, HSAN, ATFL) within the detection pipeline.

YOLO11-AU-IR tackles the challenge of detecting small UAVs in infrared imagery while ensuring computational efficiency for edge deployment. The framework employs a streamlined architecture with four key innovations, as shown in (Fig 4).

The processing pipeline begins with infrared image preprocessing (normalization and augmentation) followed by feature extraction through the YOLO11n backbone. Our contributions enhance this base architecture at three critical stages:

1. EADown Modules: Deployed in the neck at feature fusion stages, these modules preserve fine-grained features during multi-scale aggregation through dual-branch processing and targeted regularization.
2. HSAN Module: Integrated into the backbone to replace standard feature extraction modules, HSAN enhances multi-scale representation through grouped convolutions and joint channel-spatial attention, improving feature discrimination for small UAVs.
3. ATFL Loss: Applied during training, this adaptive loss function dynamically balances focus between challenging small targets and abundant background regions, improving model convergence and final performance.

The enhanced features pass through the segmentation head to produce pixel-level masks for detected UAVs. This design achieves our trilemma goals: high precision (97.7% mAP@0.50), real-time speed (59.8 FPS), and edge robustness (4.42 MB model size).

### 4.3. Efficient adaptive downsampling

In scenarios involving small-scale infrared UAV detection datasets, models frequently encounter challenges such as performance degradation and over-fitting. To address these issues, we propose the EADown, which achieves network light-weighting while preserving critical features through diversified feature extraction and efficient downsampling strategies. The core concept of EADown involves combining preliminary average pooling with dual-branch processing of input feature maps to balance global and local feature retention. Specifically, an initial 2×2 average pooling operation is applied to the input feature map to smooth feature distribution and suppress noise interference; subsequently, the feature map is equally divided along the channel dimension into two parts, each entering a different branch for downsampling and convolution operations, thereby capturing multi-scale, multi-type critical information. Global regularization would significantly impact accuracy, therefore we implemented regularization only at specific locations within the module, ensuring optimal performance while maintaining effective feature extraction.

EADown optimizes downsampling by applying average pooling followed by dual-branch convolutions. Compared to standard strided convolution downsampling, EADown reduces computational cost from 34.49 GFLOPs to 21.54 GFLOPs (37.5% reduction) while maintaining superior feature preservation for small targets (Table 3). The first branch uses a 3 × 3 convolution to capture medium-scale semantic features, while the second branch applies maximum pooling followed by 1 × 1 convolutions to preserve fine-grained local features. This combination helps retain critical small target details that might otherwise be lost during standard downsampling operations. The resulting feature maps are concatenated and passed on to HSAN for further refinement in multi-scale feature fusion.

The feature maps output from both branches are concatenated along the channel dimension, forming a comprehensive representation that includes both global and local semantics. To stabilize training and suppress over-fitting, the concatenated features undergo batch normalization (BatchNorm) and two-dimensional random deactivation (Dropout2d) processing. BatchNorm reduces feature distribution differences between different batches, accelerating model convergence; Dropout2d randomly deactivates certain channels, reducing model dependency on specific features, particularly suitable for small-scale datasets to enhance generalization capability.

Let $X\in R^{C_{1}\times H\times W}$ represent the input feature map, where $C_{1}$ denotes the number of input channels, and H and W represent height and width, respectively. The processing flow of EADown can be summarized as follows:

1. Preliminary average pooling:

$$X_{avg}=AvgPool_{2\times 2}(X)$$ (1)

2. Channel division:

$$X_{1},X_{2}=\text{Chunk}\left(X_{avg},2\right),$$ (2)

3. Branch one (3×3 convolution, downsampling):

$$Y_{1}=Conv_{3\times 3}\left(X_{1};stride=2,padding=1\right),$$ (3)

4. Branch two (3×3 maximum pooling + 1×1 convolution):

$$Y_{2}=Conv_{1\times 1}\left(MaxPool_{3\times 3}\left(X_{2};stride=2,padding=1\right)\right),$$ (4)

5. Feature concatenation and regularization:

$$Y=\text{Concat}\left(Y_{1},Y_{2}\right),Y^{\prime }=\text{Dropout2d}\left(\text{BatchNorm}(Y)\right).$$ (5)

In summary, EADown first smooths the input feature map X, then implements diversified downsampling through dual branches, and finally inputs the fused features $Y^{\prime }$ into subsequent networks, thereby maximizing critical information retention while reducing resolution. Compared to traditional single-path downsampling (such as pure convolution or pure maximum pooling), EADown preserves richer feature representations while reducing resolution through multi-branch and regularization strategies, particularly advantageous for small target detection. Features output after EADown processing will more tightly interface with subsequent HSAN to ensure effective transmission of small target details in deep networks.

### 4.4. Hetero scale attention network

When detecting UAVs of different sizes in infrared images, traditional methods often miss small or distant targets because they cannot effectively combine information from different scales [46–47]. To solve this problem, we developed the HeteroScale Attention Network (HSAN).

HSAN addresses a fundamental limitation in infrared UAV detection: small UAVs may appear as tiny heat signatures of just a few pixels, while larger UAVs occupy substantial image regions. By replacing the computationally intensive C2PSA module (129.7 GFLOPs) with our efficient HSAN design (29.29 GFLOPs), we achieve a 77.4% reduction in computational overhead while enhancing multi-scale feature fusion capability (Table 3). Each requires different feature extraction strategies. HSAN works like having multiple specialized detectors running in parallel—some focused on finding tiny thermal spots, others on larger heat patterns—then intelligently combining their findings for robust detection across all scales.

The key innovation lies in how HSAN processes features. Unlike CBAM’s sequential channel-spatial attention or YOLOv7-tiny’s polarization attention that treat all scales uniformly, HSAN’s grouped multi-scale convolutions (1 × 7 & 7 × 1–1 × 21 & 21 × 1 kernels) specifically target the multi-scale nature of infrared UAV thermal signatures. Rather than treating all image features uniformly, it divides them into groups and processes each group with different receptive fields. This allows the network to simultaneously look for both the subtle heat signature of a distant micro-drone and the more prominent thermal pattern of a nearby UAV. After extracting these multi-scale features, HSAN applies attention mechanisms to emphasize the most relevant information while suppressing background noise and interference.

Formally, the input feature map $x\in R^{B\times C\times H\times W}$ is processed through two successive 1×1 grouped convolutions to transform the channel dimension from $Ctoc_{2}$ while preserving inter-group isolation:


$$x^{\prime }=\text{Conv2d}\left(x;1\times 1,\text{groups}=g\right)\to \text{Conv2d}\left(x^{\prime };1\times 1,\text{groups}=g\right),$$ (6)

where g is the number of groups (default = 4), and each group contains $\frac{c_{2}}{g}$ channels. The resulting feature is reshaped into $x_{\text{grouped}}\in R^{B\times g\times \frac{c_{2}}{g}\times H\times W}.$ This grouping strategy divides the large channel dimension into multiple subspaces, enabling each group to independently learn distinct feature representations. This reduces interference during subsequent multi-scale and attention computations and lowers the overall computational cost.

For each group $g_{i}$, HSAN employs a multiscale convolution module to capture spatial information across varying receptive fields. The process is as follows:

First, a 5×5 depthwise-separable convolution extracts local contextual information within the group. Compared to standard convolutions, depthwise-separable convolutions maintain a large receptive field while significantly reducing computational complexity.

Subsequently, parallel branches with elongated kernels are sequentially applied in the following order: (1×7,7×1),(1×11,11×1),(1×21,21×1). This sequential processing further extends the receptive field and extracts multi-scale information. Finally, a 1×1 convolution fuses the features from all scales, yielding an enhanced group feature $g_{i}^{\prime }$.

This multi-scale strategy effectively overcomes the limitations of traditional self-attention mechanisms, which struggle to model long-range dependencies under single-scale or fixed receptive field constraints.

Following multi-scale convolution, the group features undergo channel attention to emphasize critical channels and suppress background interference. The channel attention is formulated as:


$$A_{c}\left(g_{i}^{\prime }\right)=\sigma \left(W_{2}\left(\text{ReLU}\left(W_{1}\left(\text{AvgPool}\left(g_{i}^{\prime }\right)+\text{MaxPool}\left(g_{i}^{\prime }\right)\right)\right)\right)\right)$$ (7)

where AvgPool(·) and MaxPool(·) denote global average pooling and global max pooling, respectively, $W_{1}$ and $W_{2}$ are learnable 1×1 convolutions with a reduction ratio of 16 (by default), and σ(·) is the sigmoid activation function. By combining the results of global average and max pooling, processing them through a two-layer perceptron, HSAN adaptively assigns weights to channels, enhancing those critical for UAV detection while suppressing noise or background-related channels.

Next, spatial attention is applied independently along the height and width dimensions. For the height direction, the group feature $g_{i}^{\prime }$ is pooled across the channel dimension using average and max operations, and the concatenated results are processed by a 7×7 convolution to generate a height attention map $A_{h}$. The width attention map $A_{w}$ is computed similarly, with the same kernel shape and padding. The combined attention is then applied as:


$$F_{i}=g_{i}^{\prime }⊙A_{c}\left(g_{i}^{\prime }\right)⊙A_{h}⊙A_{w},$$ (8)

where ⊙ denotes element-wise multiplication. This process enables HSAN to focus on discriminative feature dimensions at the channel level while highlighting key spatial regions along both height and width, thereby improving sensitivity to small targets and robustness against complex backgrounds.

After applying multi-scale convolution, channel attention, and spatial attention, the processed features from all groups are stacked and concatenated along the channel dimension to form the final output $x^{\prime }$. When initialized with $\text{shortcut}=\text{True}andc_{1}=c_{2}$, a residual connection adds the input feature x to the output $x^{\prime }$:


$$x_{\text{HSAN}}=x^{\prime }+x.$$ (9)

This residual connection stabilizes gradient flow and preserves information from early features, which is particularly beneficial for training on small-scale datasets. Experimental results demonstrate that replacing the conventional context-aware modules with HSAN significantly improves precision and robustness in IR UAV detection tasks, with minimal impact on inference speed and resource consumption.

### 4.5. Computational efficiency analysis

A critical consideration for edge deployment is the computational efficiency of each architectural component. Table 3 presents a detailed comparison of computational costs between our proposed modules and the modules they replace in the original YOLO11n architecture, with all measurements conducted using fixed input/output dimensions of 1024 channels to ensure fair comparison.

The computational savings are achieved through architectural optimizations: EADown’s dual-branch design with grouped operations requires fewer computations than standard strided convolution, while HSAN’s grouped convolutions and simplified attention mechanism dramatically reduce the computational burden compared to C2PSA’s complex cross-stage partial connections. These module-level optimizations contribute to the overall model efficiency, with YOLO11-AU-IR requiring only 9.0 GFLOPs compared to 10.2 GFLOPs for YOLO11n-seg, enabling real-time inference on resource-constrained edge devices while maintaining superior detection accuracy.

### 4.6. Adaptive threshold focus loss function

In infrared UAV detection, a critical challenge emerges from extreme class imbalance: UAV pixels typically represent less than 0.1% of an image, while background pixels comprise over 99.9%. Standard loss functions become overwhelmed by this abundance of easy-to-classify background pixels, causing models to achieve high accuracy by simply predicting “no UAV” everywhere—completely missing the rare but crucial UAV targets.

Our proposed Adaptive Threshold Focal Loss (ATFL) solves this problem by intelligently adjusting how much attention the model pays to different samples during training. Think of ATFL as a dynamic teacher that changes its focus based on student progress. Early in training, when the model struggles to find any UAVs, ATFL aggressively ignores easy background pixels to help the model discover these rare targets. As the model improves and begins detecting UAVs reliably, ATFL gradually shifts attention to refining difficult cases—those ambiguous pixels at UAV boundaries or partially occluded targets that could be either UAV or background.

Recent work by Muksimova et al. [48] demonstrats similar challenges in UAV-based wildfire detection, where thermal targets in IR imagery require adaptive approaches to handle extreme variations in heat signatures and background complexity. Their findings align with our observations that static loss functions fail to accommodate the temporal dynamics inherent in IR detection tasks. Standard Focal Loss [49] attempts to address class imbalance by applying a modulating factor ${\left(1-p_{t}\right)}^{\gamma }$ to reduce the influence of easy samples. However, in IR scenarios, a fixed γ value proves inadequate: early training requires aggressive down-weighting of abundant background pixels (high γ), while later stages need careful balance to avoid over-suppressing genuinely difficult small UAVs (lower γ).While YOLOv8’s DFL and VFL improve class imbalance handling through distribution-based predictions, they maintain fixed hyperparameters. Building on this foundation, ATFL introduces dynamic adaptation. The formulation begins with the standard Binary Cross-Entropy (BCE) loss:


$$L_{\text{BCE}}=-\left[y\log(p)+(1-y)\log(1-p)\right],$$ (10)

where p is the predicted probability, and p is the ground-truth label. Define p as the probability aligned with the target class:


$$p_{t}=\left\{ \begin{array}{c} p,ify=1\\ 1-p,ify=0 \end{array}\right.$$ (11)

simplifying the BCE loss to:


$$L_{\text{BCE}}=-\log\left(p_{t}\right),$$ (12)

Focal Loss extends this by introducing:


$$L_{\text{FL}}\left(p_{t}\right)=-{\left(1-p_{t}\right)}^{\gamma }\log\left(p_{t}\right),$$ (13)

where (γ>0) down-weights easy samples $\left(p_{t}\approx 1\right)$ and emphasizes hard samples $\left(p_{t}\approx 0\right)$. Yet, in highly imbalanced IR small-target detection, this uniform modulation may excessively reduce the loss of hard samples, impeding learning of critical features.

To address this, we first consider a Threshold Focal Loss (TFL):


$$L_{TFL}(p_{t})=\left\{ \begin{array}{c} -\lambda {\left(1-p_{t}\right)}^{\gamma }log(p_{t}),p_{t}\leq 0.5,\\ -\lambda {\left(1-p_{t}\right)}^{\eta }log(p_{t}),p_{t}>0.5, \end{array}\right.$$ (14)

where λ>1 amplifies the loss for hard samples $\left(p_{t}\leq 0.5\right)$, and ${\left(1-p_{t}\right)}^{\eta }$ reduces it for easy samples $\left(p_{t}>0.5\right)$, with γandη as hyper-parameters. While effective, ATFL’s fixed threshold and static parameters limit its flexibility across training dynamics and datasets.

ATFL overcomes these constraints by adaptively tuning γ and ηbased on training statistics. We compute an exponentially smoothed estimate of the average predicted probability, $\hat{p_{c}}$, over epochs:


$$\hat{{p_{c}}^{t}}=0.05\cdot p_{t}+0.95\cdot \hat{{p_{c}}^{t-1}},$$ (15)

where $p_{t}$ is the average predicted probability for the positive class across all samples in the current epoch t, and $\hat{{p_{c}}^{t-1}}$ is the prior epoch's estimate, initialized appropriately (e.g., at 0.5). The coefficients 0.05 and 0.95 balance current and historical contributions.

**Algorithm 1: Adaptive threshold focal loss**

**Input:** mini-batch {(xi,yi)}, running avg ${\text{P}}_{\text{t}1},\lambda$

**Output:** mean loss L

1: ${\text{p}}_{\text{t}}$ ← average predicted probability for positive class

2: $\hat{{{\text{p}}_{\text{c}}}^{\text{t}}}$ ← $\text{0.05×}{\text{p}}_{\text{t}}\text{+0.95×}\hat{{{\text{p}}_{\text{c}}}^{\text{t-1}}}$

3: γ ← $\text{-ln}\left(\hat{{{\text{p}}_{\text{c}}}^{\text{t}}}\right)$

4: η ← $\text{-ln}\left({\text{p}}_{\text{t}}\right)$

5: for each sample i in batch do

6:   ${\text{p}}_{\text{i}}$ ← model prediction for ${\text{x}}_{\text{i}}$

7:   ${\text{p}}_{\text{t,i}}$ ← ${\text{y}}_{\text{i}}\text{·}{\text{p}}_{\text{i}}$ + 

8:   if ${\text{p}}_{\text{t,i}}\leq \text{0.5}$ then

9:    ${\text{L}}_{\text{i}}$ ← $-\lambda {\left(\text{1-}{\text{p}}_{\text{t,i}}\right)}^{\gamma }\text{log}\left({\text{p}}_{\text{t,i}}\right)$

10:  else

11:   ${\text{L}}_{\text{i}}$ ← $-\lambda {\left(\text{1-}{\text{p}}_{\text{t,i}}\right)}^{\eta }\text{log}\left({\text{p}}_{\text{t,i}}\right)$

12:  end if

13: end for

14: L ← 

15: return L

We then define:


$$\gamma =-\ln\left(\hat{p_{c}}\right),\;\eta =-\ln\left(p_{t}\right),$$ (16)

where the natural logarithm ensures positive, increasing values as $\hat{p_{c}}$ and $p_{t}$ decrease, amplifying focus on harder samples. In early training, low $p_{t}$ yields large γ and η, prioritizing all samples. As $p_{t}$ rises, η decreases, diminishing easy-sample weights, while γ tracks long-term trends via $\hat{p_{c}}$, sustaining emphasis on hard samples.

The final ATFL is:


$$L_{ATFL}(p_{t})=\left\{ \begin{array}{c} -\lambda {\left(1-p_{t}\right)}^{\gamma }log(p_{t}),p_{t}\leq 0.5,\\ -\lambda {\left(1-p_{t}\right)}^{\eta }log(p_{t}),p_{t}>0.5, \end{array}\right.$$ (17)

with γ and η updated per epoch. The threshold of 0.5 aligns with standard binary classification conventions.

ATFL’s adaptive nature makes it particularly effective for infrared UAV detection. By automatically adjusting focus throughout training, it ensures the model first learns to find UAVs despite overwhelming background pixels, then progressively refines its ability to handle challenging cases like partially occluded targets or UAVs at thermal crossover points. Experiments show that ATFL, paired with our EADown and HSAN modules, enhances detection accuracy and robustness significantly.

## 5. Experiments

**Fig 5:**
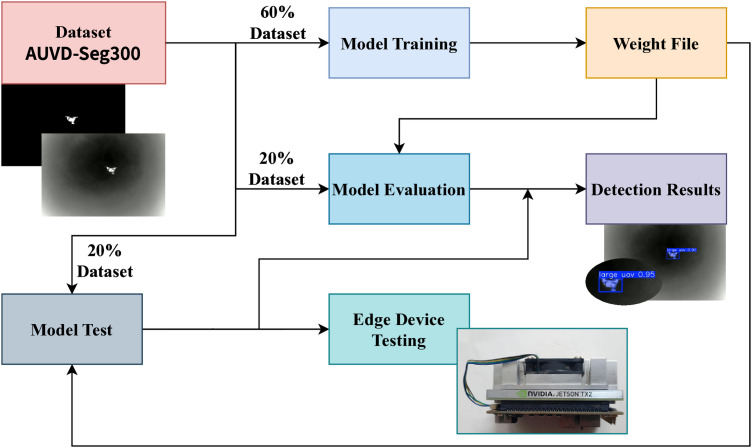
Workflow of YOLO11-AU-IR model development and testing.

In this section, we present a comprehensive evaluation of the proposed YOLO11-AU-IR model for infrared UAV detection. All experiments were conducted under the same conditions, as specified in Table 4. To ensure reproducibility and enable fair comparisons, we maintained identical data partitions and model initialization parameters across all experiments, while maintaining identical data partitions and model initialization parameters. As depicted in (Fig 5), the evaluation pipeline divides the AUV-Seg300 dataset into 60% training, 20% testing, and 20% validation data. Post-training, the model's learned weights yield detection predictions, followed by rigorous quantitative analysis.

### 5.1. Dataset

To enhance model generalization capability, we implemented comprehensive data augmentation techniques during training. The augmentation pipeline includes HSV color space transformations with moderate hue adjustments (±1.5%) to accommodate thermal signature variations, significant saturation enhancement (70%) to strengthen thermal contrast, and value enhancement (40%) to simulate different infrared intensity levels. Spatial transformations were carefully calibrated with a 10% translation range to simulate UAV position variations and a scaling factor of 0.5 to introduce size variability while maintaining detection integrity. We employed horizontal flipping with a 50% probability to effectively double the training data while preserving the physical characteristics of infrared signatures. The augmentation strategy particularly emphasized mosaic composition with full probability (100%) to improve multi-object detection capability and enhance robustness against complex backgrounds. These transformations were specifically designed to address challenges posed by environmental interference while maintaining the physical consistency of thermal patterns, with all parameters empirically optimized for infrared UAV detection scenarios.

### 5.2. Evaluation metrics

We evaluated YOLO11-AU-IR to assess its performance in infrared UAV detection tasks using several key metrics. Precision (P) and Recall (R) measure detection accuracy and completeness, respectively, defined as:


$$\text{Precision}=\frac{TP}{TP+FP}$$ (18)

$$\text{Recall}=\frac{TP}{TP+FN}$$ (19)

where TP denotes true positives (correctly detected UAVs), FP denotes false positives (incorrect detections), and FN denotes false negatives (undetected UAVs). Mean Average Precision (mAP) assesses overall detection performance across Intersection over Union (IoU) thresholds, with mAP@0.5 reported at IoU = 0.5 and mAP@0.5:0.95 averaged over IoU from 0.5 to 0.95 in 0.05 increments for a more rigorous evaluation. Inference Speed, measured in frames per second (FPS), ensures suitability for real-time applications. Model size, quantified by parameter count and storage requirements (MB), confirms the model's efficiency for embedded systems. These metrics collectively evaluate YOLO11-AU-IR across detection accuracy, completeness, overall performance, real-time efficiency, and resource utilization.

We acknowledge that our evaluation focuses on traditional mAP metrics without boundary-specific assessments. While these metrics effectively measure overall detection and segmentation performance, boundary-aware metrics such as Boundary IoU or Boundary F1 would provide additional insights into fine-grained segmentation quality, particularly crucial for small infrared UAV targets where precise boundary delineation affects threat assessment. This represents a limitation of our current evaluation framework and constitutes valuable future work.

### 5.3. Comparative experiments

To comprehensively validate the effectiveness of YOLO11-AU-IR for infrared UAV detection, we conducted extensive comparative evaluations against mainstream lightweight segmentation models. All models were trained under identical hardware and software environments using the AUVD-Seg300 dataset with consistent training parameters to ensure fair comparison.

**Fig 6:**
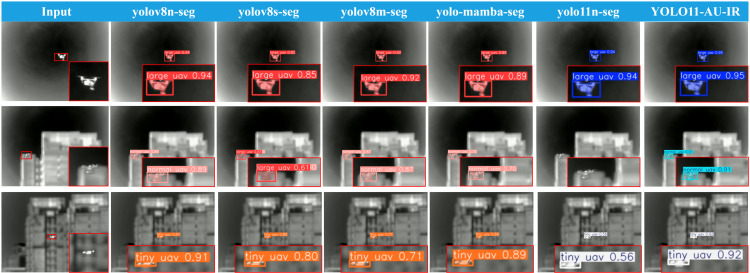
Input images and segmentation results of various models on the AUVD-Seg300 dataset.

The detection performance comparison in (Fig 6) shows that although all models achieved satisfactory results in normal environments, their capabilities diverged substantially when facing challenging scenarios, especially those involving minimal lighting or small targets at long ranges.For low-contrast “tiny UAV” targets, YOLO11-AU-IR demonstrated more stable and precise boundary delineation while effectively suppressing background noise, whereas other models (e.g., YOLO11n-seg, YOLOv8n-seg) occasionally produced false negatives or lower confidence detections in identical scenarios.

The quantitative results presented in Tables 5a and 5b reveal YOLO11-AU-IR’s substantial performance advantages across all evaluation metrics. As shown in Table 5a, among lightweight models suitable for edge deployment, our model with only 2.14M parameters achieves 97.7% mAP@0.50 and 75.2% mAP@0.50:0.95, representing significant improvements over competitive lightweight baselines. Notably, YOLO11-AU-IR exhibits exceptional inference stability with 59.8 ± 0.74 FPS, demonstrating the lowest variance among all tested models—a critical characteristic for reliable real-world deployment.

While medium and heavyweight models in Table 5b achieve competitive performance, they require substantially more computational resources. For instance, YOLOv8m-seg achieves slightly higher mAP scores (97.9% and 74.9%) but requires 52.2MB storage—over 11 × larger than YOLO11-AU-IR—making it unsuitable for edge deployment. This comparison highlights the superior accuracy-efficiency balance achieved by our approach, particularly crucial for resource-constrained applications.

**Fig 7:**
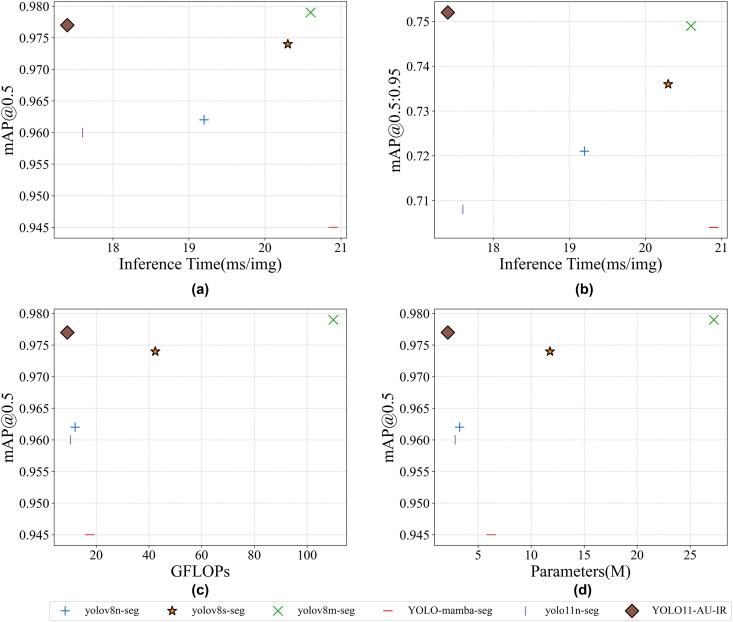
Model efficiency and performance trade-off analysis. **(a)** Relationship between mAP@0.5 and inference time; **(b)** Relationship between mAP@0.5: 0.95 and inference time; **(c)** Relationship between mAP@0.5 and GFLOPs; **(d)** Relationship between mAP@0.5 and the number of parameters.

The efficiency-performance trade-off analysis in (Fig 7) reveals YOLO11-AU-IR's competitive advantage, with its positioning in the optimal upper-left region of the performance-speed space outperforming alternative models in balancing computational efficiency and detection capability. Our model delivers peak detection accuracy at the lowest computational cost among competitive solutions, requiring only 9.0 GFLOPs—an 11.8% reduction from the baseline YOLO11n-seg. This efficiency stems directly from our architectural optimizations, where EADown achieves 37.5% computation reduction and HSAN provides 77.4% efficiency improvement over replaced modules. The parameter efficiency analysis further validates our design choices, with YOLO11-AU-IR requiring 24.5% fewer parameters than YOLO11n-seg and 34.3% fewer than YOLOv8n-seg while maintaining superior performance.

**Fig 8:**
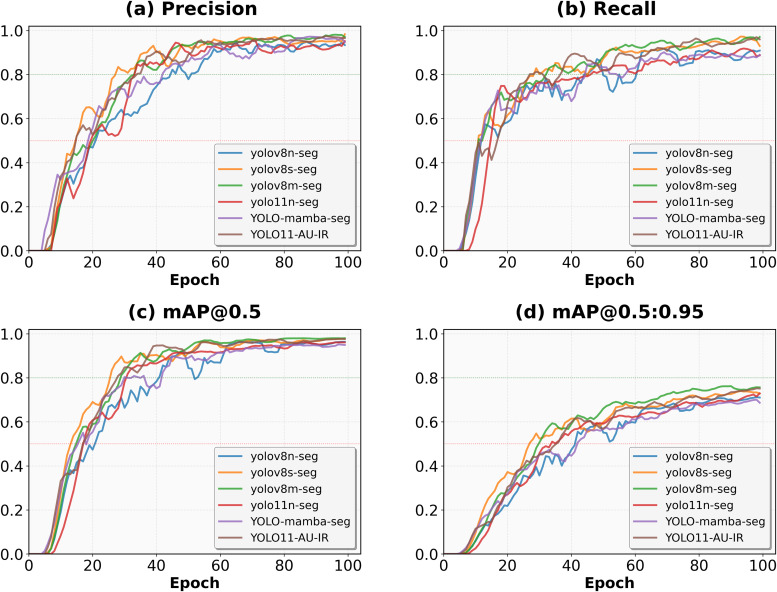
Comparison of training performance of different models on the AUVD-Seg300 dataset. **(a)** Accuracy curve; **(b)** Recall curve; **(c)** mAP@0.50 curve; **(d)** mAP@0.50:0.95 curve.

The training dynamics illustrated in (Fig 8) demonstrate that YOLO11-AU-IR achieves superior convergence speed and stability compared to baseline models. The model achieves remarkably faster convergence, reaching 90% of final performance by epoch 40—approximately 20 epochs earlier than baseline models. Throughout training, YOLO11-AU-IR maintains exceptional stability with minimal oscillations in precision and recall curves, contrasting with the ± 5–10% fluctuations observed in YOLOv8n-seg and YOLO-mamba-seg. This stability validates the effectiveness of our Adaptive Threshold Focal Loss in managing extreme class imbalance. Most significantly, the model demonstrates sustained improvement in later training stages (epochs 80–100), gaining an additional 1% mAP@0.50:0.95 when other models plateau, suggesting that our architectural innovations enable continued feature refinement even in advanced training phases.

Further analysis from a multi-scale target detection perspective (Table 6) reveals that YOLO11-AU-IR maintains statistically superior performance across all UAV type categories. This categorization is particularly relevant for security applications, as each UAV type presents distinct detection challenges: large UAVs have stronger thermal signatures but may operate at greater distances, while tiny UAVs are inherently difficult to detect due to minimal heat emission and small physical size. YOLO11-AU-IR achieves 99.5% mAP@0.50 for both large and tiny UAV types, with the most significant improvement in tiny UAV detection at stricter IoU thresholds (71.8% mAP@0.50:0.95), representing a 5.4 percentage point improvement over the baseline. This demonstrates our model's effectiveness across the full spectrum of UAV threats. Although YOLOv8s-seg and YOLOv8m-seg also perform well for large UAV detection, they require significantly more computational resources (42.4 and 110.0 GFLOPs respectively) while achieving lower performance on tiny UAVs—the most challenging category for early threat detection.

**Table 6:** Comparison of experimental results for multi-scale object detection.

Model	mAP@0.50 (%)			mAP@0.50:0.95 (%)		
	Large	Normal	Tiny	Large	Normal	Tiny
**yolov5n-seg**	74.3	49.5	85.9	50.9	32.6	46.6
**yolov5s-seg**	95.1	94.1	98.1	68	67.6	56.7
**yolov5m-seg**	92.1	93.2	99.5	65.7	72.4	63.7
**yolov7-seg**	98.8	94.5	99.5	72.8	75.8	64.5
**yolov8n-seg**	97.2	92.0	99.5	71.7	74.1	70.6
**yolov8s-seg**	98.6	94.2	99.5	77.6	75.3	67.8
**yolov8m-seg**	99.3	95.0	99.5	75.8	76.4	72.5
**YOLO-mamba-seg**	94.3	92.0	97.2	69.7	73.0	68.5
**yolo11n-seg**	96.9	92.9	98.1	72.8	73.3	66.4
**YOLO11-AU-IR**	**99.5**	**94.1**	**99.5**	**77.5**	**76.3**	**71.8**

*UAV type definitions based on AUVD-Seg300 dataset categorization: Large UAV (multi-rotor platforms with 6 + rotors, > 600mm diagonal wheelbase), Normal UAV (standard quad-copters, 250–600 mm diagonal wheelbase), and Tiny UAV (micro/mini drones, < 250mm diagonal wheelbase). These categories reflect different operational capabilities and detection challenges in infrared imagery.

### 5.4. Model robustness and error analysis

To comprehensively evaluate model robustness, we performed stratified 3-fold cross-validation. While the limited dataset size (240 images per fold) introduces expected variability, our results demonstrate consistent improvements over the baseline across all folds.

As shown in Table 7, YOLO11-AU-IR demonstrates consistent improvements over the baseline. While the limited dataset size introduces variance, YOLO11-AU-IR consistently outperforms the baseline across all folds, with lower standard deviation indicating improved stability.

**Table 7:** Three-fold cross-validation performance comparison.

Metric	Model	Fold 1	Fold 2	Fold 3	Mean ± SD	Improvement
**mAP@0.50 (%)**	yolo11n-seg	88.9	95.7	95.7	93.4 ± 3.93	
	YOLO11-AU-IR	90.9	96.2	97.4	94.8 ± 3.46	+1.4%

**Fig 9:**
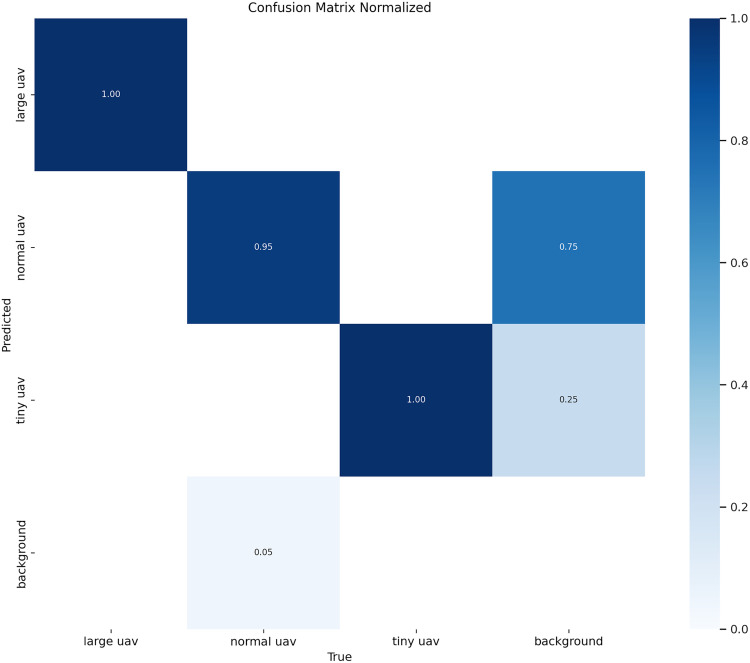
Normalized confusion matrix for YOLO11-AU-IR showing classification performance across UAV categories.

Classification Performance Analysis (Fig 9) reveals that YOLO11-AU-IR achieves exceptional detection accuracy across UAV categories. Detection of large UAVs and tiny UAVs achieves perfect normalized accuracy (100%), indicating highly reliable recognition for these classes.For normal UAVs, the classifier achieves 95% correct predictions, with 5% being mis-classified as background. Notably, contrary to earlier interpretation, normal UAVs are not mis-classified as tiny UAVs, and tiny UAVs are partially mis-classified as background (25%), suggesting some ambiguity in differentiating small UAVs from non-target thermal signatures in the IR domain. Background suppression also performs well, with only 5% of background instances incorrectly classified as normal UAVs, reflecting robust filtering of non-UAV thermal features.

To further understand the spatial distribution of these errors revealed in the confusion matrix, we conducted an error-focused Grad-CAM analysis to visualize where and why mis-classifications occur, directly addressing the spatial patterns behind the confusion matrix statistics.

**Fig 10:**
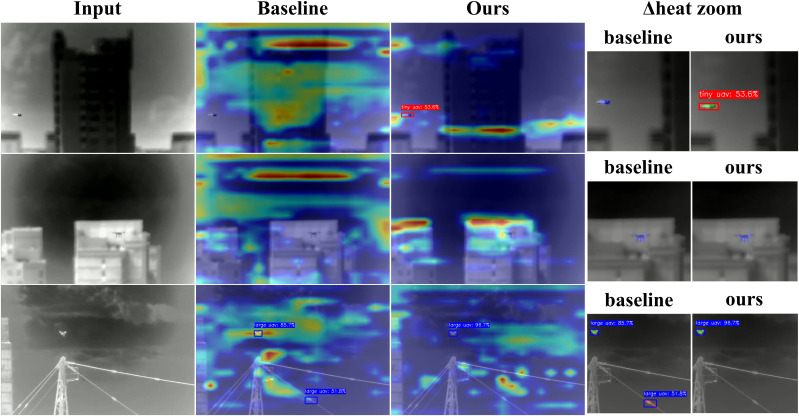
Error-focused Grad-CAM comparison.

Spatial error analysis based on Grad‑CAM visualisations (Fig. 10) reveals the underlying causes of misclassification in UAV detection. The first column shows the original infrared inputs, while the second and third columns overlay Grad‑CAM maps for the baseline and our improved model, respectively; red hues correspond to regions of high activation. The fourth column depicts a Δheat image obtained by subtracting our activations from the baseline and then zooming the error‑prone area. In this difference map, red marks activations present only in the baseline and therefore likely to cause false positives, blue marks activations missing from the baseline and thus linked to false negatives, and green marks new activations introduced by our model that coincide with true positives.

Reading the rows from top to bottom reveals how individual error modes in the confusion matrix arise and how each architectural component addresses them. In the first row, a tiny UAV blends into a textured urban background and is overlooked by the baseline because its down‑sampling path suppresses faint, sub‑pixel features; the excessive red activation on building edges reflects this confusion. Our model, equipped with the EADown module, retains these fine‑grained gradients, turning the previously blue area around the UAV into a green patch that signals a newly recovered detection. The second row illustrates a normal‑sized UAV flying close to thermally active building facades. The baseline generates scattered, scene‑wide activations that dilute its focus and lead to a five‑percent mis-classification rate. By contrast, the HSAN module adds multi‑scale attention that suppresses static, large‑area heat and tightens the focus on the genuine UAV signature, replacing diffuse red blotches with a concentrated green outline around the target. In the third row, where large UAVs are almost perfectly recognised, the baseline still allows activation to spill along cables and the transmission tower, indicating imprecise boundaries. Our Adaptive‑Threshold Focal Loss steers gradients towards pixels whose logits lie near the decision threshold, giving extra weight to boundary regions and producing a crisp contour that replaces the baseline’s spill‑over with clean segmentation; the green band around the fuselage confirms this refinement.

Taken together, these observations show that the errors visualised in the confusion matrix follow predictable spatial patterns rather than random noise: tiny‑UAV mistakes emerge when the thermal footprint approaches sensor noise, normal‑UAV errors cluster at thermal boundaries formed by infrastructure, and large‑UAV detection succeeds once boundary delineation is enforced.

### 5.5. Ablation studies

To rigorously evaluate the contribution of each proposed component, we conducted a systematic ablation study using the AUVD-Seg300 dataset. Beginning with the baseline architecture (YOLO11n-seg), we progressively incorporated our proposed modules—Efficient Adaptive Downsampling (EADown), HeteroScale Attention Network (HSAN), and Adaptive Threshold Focal Loss (ATFL)—to quantify their individual and collective impact on detection performance.

Table 8 presents the quantitative results across five model configurations. To provide clearer visualization of the progressive performance improvements, Fig 10 presents a bar chart comparing the ablation study results.

**Table 8:** Ablation study results.

Model	EADown	HSAN	ATFL	mAP@0.50 (%)	mAP@0.50:0.95 (%)
**Baseline**				96.0	70.8
**Model1**	✓			97.3	71.8
**Model2**		✓		97.2	72.1
**Model3**	✓	✓		96.9	73.0
**YOLO11-AU-IR**	✓	✓	✓	97.7	75.2

**Fig 11:**
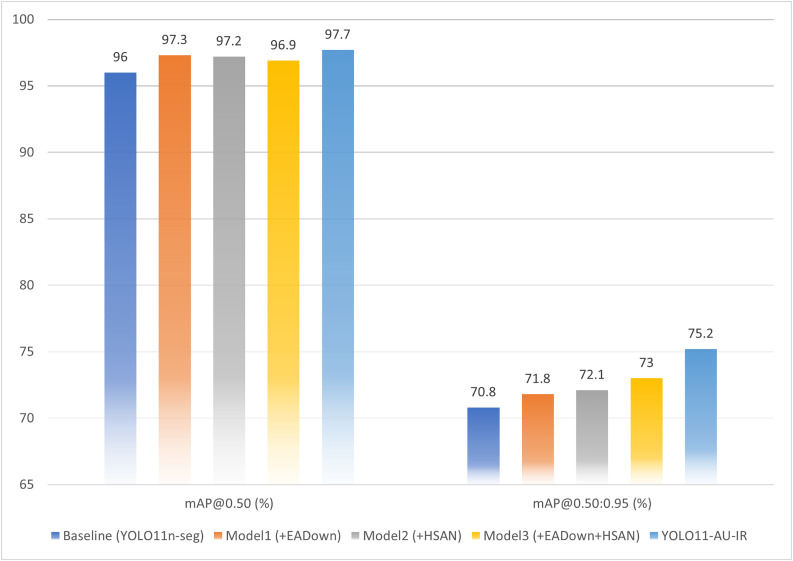
Bar chart visualization of ablation study results showing progressive performance improvements with each module integration.

**Fig 12:**
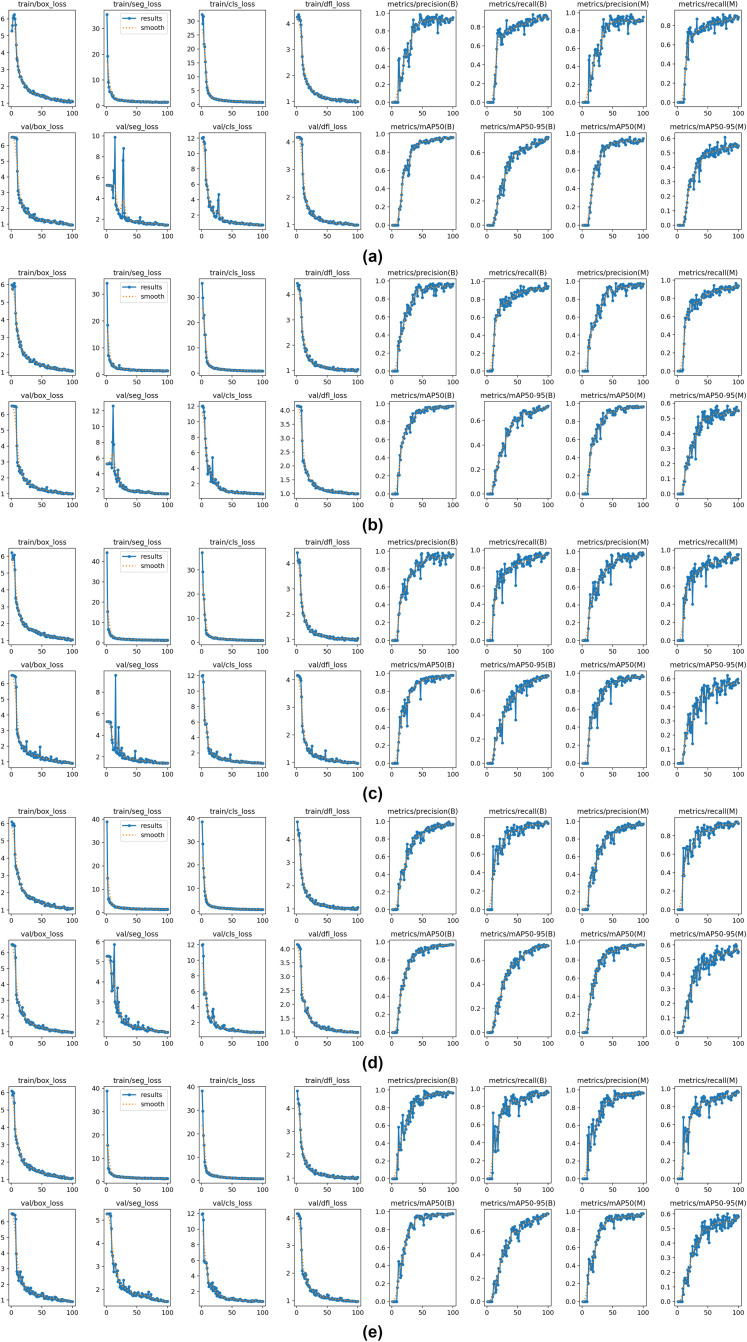
Training performance comparison across ablation configurations. **(a)** Baseline, **(b)** Baseline+EADown, **(c)** Baseline+HSAN, **(d)** Baseline+EADown+HSAN, **(e)** YOLO11-AU-IR. Each subplot shows training/validation loss curves and four key metrics: precision, recall, mAP@0.50, and mAP@0.50:0.95.

As shown in (Fig 11), the baseline model achieved 96.0% mAP@0.50 and 70.8% mAP@0.50:0.95, establishing the foundation for our comparative analysis. The bar chart reveals that while individual modules provide modest improvements in mAP@0.50 (1.2–1.3%), the more significant gains occur in the stricter mAP@0.50:0.95 metric, with the complete system achieving a 4.4% absolute improvement over the baseline. (Fig 12) illustrates the detailed training dynamics for each configuration throughout the training process.

The integration of EADown (Model1) substantially improved performance to 97.3% mAP@0.50 and 71.8% mAP@0.50:0.95, with (Fig 12) (b) revealing significantly enhanced training stability and faster convergence compared to the baseline. This improvement validates our hypothesis that preserving fine-grained features during spatial compression is essential for small target detection in infrared imagery.

Similarly, implementing HSAN alone (Model2) yielded comparable enhancements in mAP@0.50 (97.2%) with slightly superior gains in mAP@0.50:0.95 (72.1%). The training dynamics illustrated in (Fig 12) (c) demonstrate HSAN’s efficacy in addressing multi-scale feature representation—critical for accommodating the diverse target sizes encountered in UAV detection scenarios. The smoother training curves indicate that the attention mechanism effectively stabilizes the learning process.

The concurrent implementation of both EADown and HSAN (Model3) produced an intriguing performance profile: a slight reduction in mAP@0.50 to 96.9% compared to their individual implementations, but a substantial improvement in the more rigorous mAP@0.50:0.95 metric to 73.0%. This phenomenon suggests that while the combined modules might marginally compromise performance under lenient evaluation criteria, they significantly enhance localization precision under more stringent conditions—a characteristic of greater relevance for operational applications where precise boundary delineation is crucial.

The complete YOLO11-AU-IR model integrating all three components achieved optimal performance across all metrics, with 97.7% mAP@0.50 and 75.2% mAP@0.50:0.95, representing absolute improvements of 1.7% and 4.4% over the baseline, respectively. As evidenced in (Fig 12) (e), the full model exhibits remarkably stable convergence patterns with minimal oscillations in both training and validation metrics. The disproportionate improvement in mAP@0.50:0.95 compared to mAP@0.50 indicates that our approach particularly excels at precise localization across varying scales and IoU thresholds.

The ablation results reveal important architectural insights beyond mere performance metrics:

1. Complementary Module Design: The synergistic effect observed when combining all three components demonstrates their complementary nature in addressing distinct aspects of the infrared UAV detection challenge. EADown preserves spatial details, HSAN enhances multi-scale fusion, and ATFL optimizes the learning dynamics.
2. Training Stability: The progressive enhancement in training stability across configurations suggests that our proposed modules not only improve final performance but also enhance optimization behavior, resulting in more reliable and reproducible training outcomes.
3. Balanced Performance: The improvements are consistent across different evaluation metrics and target scales, indicating a comprehensive enhancement rather than selective optimization for specific scenarios.

These findings provide compelling evidence that each proposed component makes a substantive contribution to overall system performance, with their integration yielding a detection framework that effectively addresses the fundamental “tri-lemma” of precision, speed, and robustness in infrared UAV detection applications while maintaining the lightweight characteristics essential for edge deployment scenarios.

### 5.6. Edge device deployment and evaluation

**Fig 13:**
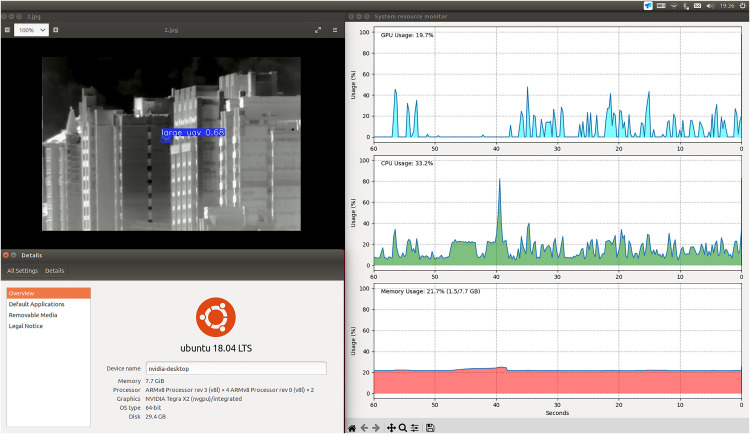
Inference testing on the NVIDIA Jetson TX2 platform.

To validate the practical utility of our model in resource-constrained environments, we deployed YOLO11-AU-IR on an NVIDIA Jetson TX2 embedded platform and conducted comprehensive inference testing. The edge device being tested is shown in (Fig 13).

**Fig 14:**
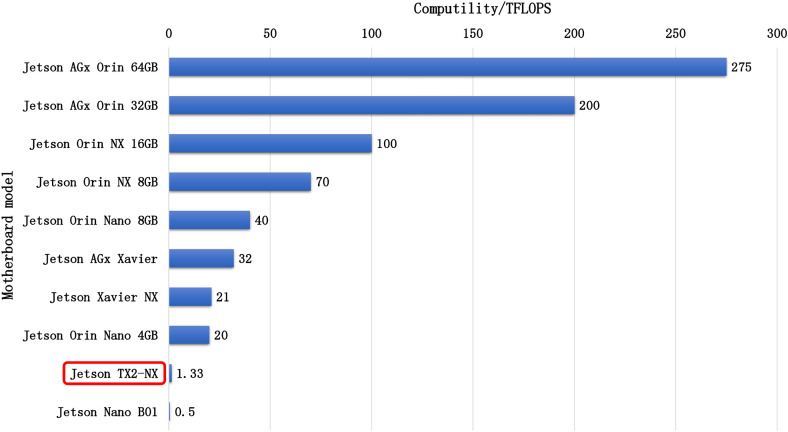
Computational performance comparison among Jetson edge devices.

The Jetson TX2 platform features a 6-core 64-bit ARM Cortex-A57 processor with a maximum frequency of 2.0352 GHz, 2 MB L2 cache, and approximately 7.7 GB total memory. It runs on Ubuntu (Fig 14), the Jetson TX2 occupies a moderate position within the Jetson family in terms of raw computational performance, measured in TFLOPS (trillions of floating-point operations per second). We specifically selected this platform to present a meaningful challenge for real-time performance in small-target infrared detection while showcasing the model's applicability across various computational environments.

Given that practical applications often require low-power, cost-effective solutions, we conducted our inference tests exclusively on the ARMv8l CPU without GPU acceleration. The software environment included NVIDIA-JetPack 4.6.6-b24, Python 3.8.0, OpenCV-Python 4.11.0.86, and PyTorch 2.0.1.

To enhance inference efficiency, we applied post-training dynamic quantization to optimize our model for edge deployment. Dynamic quantization converts model weights from FP32 to INT8 while performing on-the-fly quantization of activations during inference, providing an optimal balance between model compression and computational efficiency.

For the ONNX format, we utilized the quantize_dynamic() function from onnxruntime.quantization.quantize, using ONNX’s default configuration. This approach linearly maps floating-point values to the INT8 integer range through scale and zero point parameters, significantly reducing memory footprint and storage requirements without requiring calibration datasets.

For the TorchScript format, we employed PyTorch’s API using torch.quantization.quantize_dynamic. This function accepts the model and converts FP32 weights to INT8, while activations are converted to INT8 in real-time before computation, thereby improving computational efficiency during inference.

Both quantization approaches were applied post-training, eliminating the need for quantization-aware training or calibration datasets. The dynamic quantization strategy proved particularly effective for our edge deployment scenario, achieving substantial model size reduction while maintaining high detection accuracy. This approach is ideal for inference workloads where real-time conversion of activations provides the best balance between performance and accuracy in infrared UAV detection tasks.

Tables 9 and 10 compare inference results of YOLO11-AU-IR against YOLOv8n-seg, YOLOv8s-seg, YOLOv8m-seg, and yolo11n-seg on the AUVD-Seg300 test set for ONNX (INT8) and TorchScript (optimize, INT8), respectively. The metrics evaluated are mAP@0.50, FPS, peak CPU utilization, and maximum memory usage. Note that FPS values are generally lower for CPU-only inference, but remain informative for real-world, low-power scenarios.

**Table 9:** Model performance comparison for ONNX (INT8) deployment.

Model	mAP@0.50	FPS	Max CPU(%)	Max Mem(%)
yolov8n-seg	0.94	1.56	83.6	27.0
yolov8s-seg	0.97	0.66	83.6	28.1
yolov8m-seg	0.98	0.30	83.4	29.5
yolo11n-seg	0.91	1.61	83.2	27.1
YOLO11-AU-IR	0.95	1.55	83.5	26.9

**Table 10:** Model performance comparison for torchscript (optimize, INT8) deployment.

Model	mAP@0.50	FPS	Max CPU(%)	Max Mem(%)
yolov8n-seg	0.94	1.42	66.4	43.1
yolov8s-seg	0.97	0.52	66.8	50.1
yolov8m-seg	0.98	0.21	67.1	54.3
yolo11n-seg	0.91	1.28	67.0	36.4
YOLO11-AU-IR	0.95	1.36	67.1	38.0

In both CPU‑only deployment modes, YOLO11‑AU‑IR achieves a strong balance of accuracy and efficiency. With ONNX (INT8) it reaches an mAP@0.50 of 0.95, delivers 1.55 FPS—virtually matching the fastest baseline—and records the lowest memory usage at 26.9%. Under TorchScript (optimize, INT8) it maintains the same accuracy while running at 1.36 FPS and keeping its memory footprint below all YOLOv8 variants and close to yolo11n‑seg. These results confirm YOLO11‑AU‑IR as a competitive choice for low‑power embedded inference; Table 11 further dissects its power and memory characteristics on Jetson TX2.

**Table 11:** Module-wise resource consumption analysis on NVIDIA Jetson TX2.

Variant	Avg. mem-power (mW)	Avg. CPU-power (mW)	Avg. RAM usage (MB)
**Baseline**	1446.34	1449.62	2027.46
**+ EADown only**	1454.20 (+0.5%)	1479.00 (+2.0%)	1863.00 (−10.1%)
**+ HSAN only**	1469.37 (+1.6%)	1460.46 (+0.7%)	1894.85 (- 8.6%)
**Ours**	1457.17 (+0.8%)	1483.23 (+2.3%)	1899.75 (- 8.3%)

The resource consumption analysis reveals an excellent trade-off between computational efficiency and memory optimization. While our proposed modules introduce minimal power overhead—with memory power increasing by only 0.8% and CPU power by 2.3%—they achieve a substantial 8.3% reduction in memory footprint. This characteristic is particularly advantageous for edge deployment scenarios where memory constraints are typically more critical than power budgets.

The module-wise breakdown shows that EADown contributes most to memory savings due to its efficient dual-branch design that eliminates redundant feature storage. HSAN, while introducing slightly higher power consumption due to its multi-scale attention computations, still maintains an 8.6% memory reduction through its grouped convolution strategy. The combined implementation achieves a balanced profile, demonstrating that our architectural optimizations successfully reduce memory pressure without significantly impacting power efficiency—a crucial consideration for battery-powered edge devices in continuous surveillance applications.

Given the complexity of detecting small, low-contrast UAVs in infrared imagery, YOLO11-AU-IR’s 1.5 FPS performance on a CPU is both commendable and sufficient for various real-world applications where frame rates above 1 FPS are acceptable. In many scenarios (e.g., slow or moderate UAV speeds), this inference rate provides ample capacity for timely threat detection and responsive intervention.

Moreover, the model's small memory footprint (26.9% in ONNX and 38.0% in TorchScript) underscores its suitability for resource-limited edge devices. In tandem with its high accuracy and balanced CPU usage, these attributes confirm YOLO11-AU-IR’s feasibility as a low-power, cost-effective solution for infrared UAV detection in settings where GPU acceleration may be unavailable. The consistency between ONNX and TorchScript results further highlights the model's adaptability and robustness across different optimization pipelines and hardware configurations.

## 6. Conclusion

YOLO11-AU-IR successfully addresses the infrared UAV detection tri-lemma through three purpose-built innovations: HSAN’s grouped multi-scale convolutions for thermal signatures at varying scales, ATFL’s epoch-adaptive loss weighting for evolving sample difficulty, and EADown’s dual-branch downsampling for small-target preservation. These technical contributions, distinctly different from general-purpose optimizations in existing frameworks, enable our model to achieve 97.7% mAP@0.50 and 75.2% mAP@0.50:0.95 while maintaining real-time performance and edge deploy-ability.

Despite these achievements, we acknowledge several limitations in the spirit of transparent scientific reporting. First, the AUVD-Seg300 dataset, while providing crucial pixel-level annotations for infrared UAV segmentation, is limited to 300 images derived from a single source. This constraint may affect the model's generalization to infrared sensors with different characteristics or diverse operational environments. Second, the lack of suitable external infrared UAV segmentation datasets prevented comprehensive cross-dataset validation—a challenge stemming from the nascent state of pixel-level infrared small-target segmentation research. Third, our edge deployment evaluation, though demonstrating feasibility, revealed that CPU-only inference at 1.5 FPS may be insufficient for tracking high-speed UAVs. Fourth, our evaluation relies on standard mAP metrics without incorporating boundary-aware assessments, which could provide deeper insights into segmentation precision for small thermal targets. Fifth, the present evaluation is restricted to frame-level benchmarks and thus cannot quantify temporal consistency across continuous video or performance under night-to-day illumination shifts. Finally, while our ablation studies validate each component’s contribution, the relatively small dataset size limits the statistical power of our cross-validation results.

Future work will focus on expanding the dataset through multi-source acquisition, establishing standardized benchmarks for infrared small-target segmentation, exploring hardware acceleration for enhanced edge performance, incorporating boundary-aware evaluation metrics, and investigating multi-sensor fusion with adversarial robustness testing. By releasing our code and dataset, we aim to catalyze further research in this critical domain of infrared UAV detection.
